# “Real-Time” High-Throughput Drug and Synergy Testing for Multidrug-Resistant Bacterial Infection: A Case Report

**DOI:** 10.3389/fmed.2018.00267

**Published:** 2018-09-20

**Authors:** Wei Sun, Shayla Hesse, Miao Xu, Richard W. Childs, Wei Zheng, Peter R. Williamson

**Affiliations:** ^1^National Center for Advancing Translational Sciences, Bethesda, MD, United States; ^2^Laboratory of Molecular Biology, National Cancer Institute, Bethesda, MD, United States; ^3^Transplantation Immunotherapy, Hematology Branch, National Heart Lung and Blood Institute, Bethesda, MD, United States; ^4^Laboratory of Clinical Infectious Diseases, National Institute of Allergy and Infectious Diseases, National Institutes of Health, Bethesda, MD, United States

**Keywords:** multidrug-resistant organisms, *Klebsiella pneumoniae*, real-time drug test, antibiotic combination therapy, drug repurposing screen

## Abstract

Antibiotic management of infections with multidrug-resistant organisms (MDRO) represents a complex clinical challenge. We report here the first patient with a severe MDRO infection managed with assistance of a novel “real-time” 3-day high-throughput screen (HTS) that allowed screening of 9 drugs in 14 combinations in 2,304 total samplings. Identified synergies were used to modify patient therapy with the goal of reducing drug-induced toxicity. The desired clinical outcome was achieved on the HTS-informed therapeutic regimen, supporting the utility of HTS technology to expand standard antimicrobial susceptibility testing.

## Background

Infections with multidrug-resistant organisms (MDRO) have emerged as a worldwide health crisis with approximately two million cases and 23,000 deaths in the U.S. annually (1). Rapid identification of effective drugs is key to patient outcome (2). However standard susceptibility testing by broth microdilution, disk diffusion, gradient diffusion and traditional automated instrument systems are suitable for testing only ~20 antibiotics with limited capacity for testing drug combinations, despite routine use of combination therapy in these situations (3). Recently, while performing high-throughput (HTS) antibiotic screening for MDROs in the research setting, we encountered a severely ill patient with a complex MDRO infection. We successfully applied this highly-automated quantitative technology in miniaturized 1,536-well plate format (4) to patient-derived bacterial isolates and obtained results within a clinically-actionable time frame (in our case, a 3-day turnaround time for the primary drug screen). While this approach does not replace traditional microbiological laboratory methods, the results exemplify how HTS can rapidly identify alternative patient-specific drug combinations, providing empiric data on a wide range of theoretical treatment options for these more challenging MDRO cases.

In the primary compound screen, the bacterial strains (KP11, Ec1A, and Ec2B) were prepared and antibacterial growth assays were performed as previously described (4). Briefly, 2.5 μl TSB medium was dispensed into each well of 1,536-well plates followed by addition of 23 nl compound and 2.5 μl/well of bacterial culture with a final dilution of 1:500. After incubation for 7 h at 37°C, 5% CO2, the plates were measured for absorbance (OD600). The lead compounds were confirmed using the broth microdilution assay. The lead compounds against KP11 were also evaluated in a broth microdilution analysis according to the methods recommended by the CLSI. Briefly, a standardized inoculum was prepared by diluting the overnight culture to an optical density 625 nm (OD625) of 0.1 (equivalent to a 0.5 McFarland standard). The suspended inoculum at 1 × 10^6^ colony forming units per milliliter (CFU/ml) in 100 μl was added into each well of a 96-well plate containing 100 μl of test compound in Mueller Hinton Broth (MHB). The plates were incubated for 24 h at 37°C and microbial growth with each test compound and combination was determined by measuring the optical density at 625 nm and visually by scoring the plates ± for bacterial growth.

## Case report

The patient was a 16 year-old Kenyan male with severe aplastic anemia resulting in transfusion dependence. He sought care in India where he was treated with horse anti-thymocyte globulin (h-ATG) and cyclosporine. The patient was unresponsive to treatment and had several hospitalizations for disease-related complications. During this time he accumulated multiple risk factors for MDRO carriage including frequent antibiotic and healthcare exposure. He was transferred to the NIH for enrollment in a research study involving a potential haplo-cord transplant, but arrived septic with vancomycin-resistant *Enterococcus fecium* (VRE) and MDR *E. coli*-positive blood cultures. The source was identified as a large superinfected presacral hematoma, thought to have resulted from chronic rectal tube trauma. Given the patient's pressor requirement, severe pancytopenia and advanced debilitation, deep surgical resection of the infected hematoma was deemed impractical. The clinical strategy shifted to gaining sufficient control of the infection to enable hematopoietic reconstitution via stem cell transplant.

Expanded susceptibility testing for the two MDR *E. coli* isolates from the blood showed highly resistant organisms with *in vitro* susceptibility to colistin/polymyxin B and tigecycline only. Borderline susceptibility to imipenem was detected in one of the two isolates. Three MDR isolates detected on peri-rectal screening (one isolate of *Klebsiella pneumoniae*, two isolates of *E. coli*) showed susceptibility to colistin/polymyxin B, tigecycline and ceftazidime-avibactim. The VRE isolate showed susceptibility to daptomycin and linezolid. Consequently, the patient was treated with an antibiotic regimen that included daptomycin, imipenem, ceftazidime-avibactim, colistin and tigecycline. This combination was formulated to accommodate the differing antibiotic sensitivities among the gram-negative isolates and to apply aggressive pressure to a large inoculum of polymicrobial MDROs poised to continue seeding the patient's bloodstream. In this setting of extensive rectal fistulization and severe immunocompromise, antifungal therapy was added empirically.

Following initiation of antibiotics the patient's fever defervesced, his blood cultures cleared and his hemodynamic instability improved. However, a direct hyperbilirubinemia developed between Week 1 and Week 3 of admission, reaching a peak total bilirubin level of 26.3 mg/dl. Transaminases remained normal. No signs of cholestatic obstruction were observed by imaging. There was concern that imipenem may be contributing to cholestasis as has been reported previously for beta-lactam antibiotics, particularly carbapenems (5). However, there was also concern that discontinuation of imipenem could compromise control of the patient's MDROs. The preservation of hepatic function being imperative, it was decided that the patient would be trialed on a carbapenem-sparing regimen.

**Figure 1:**
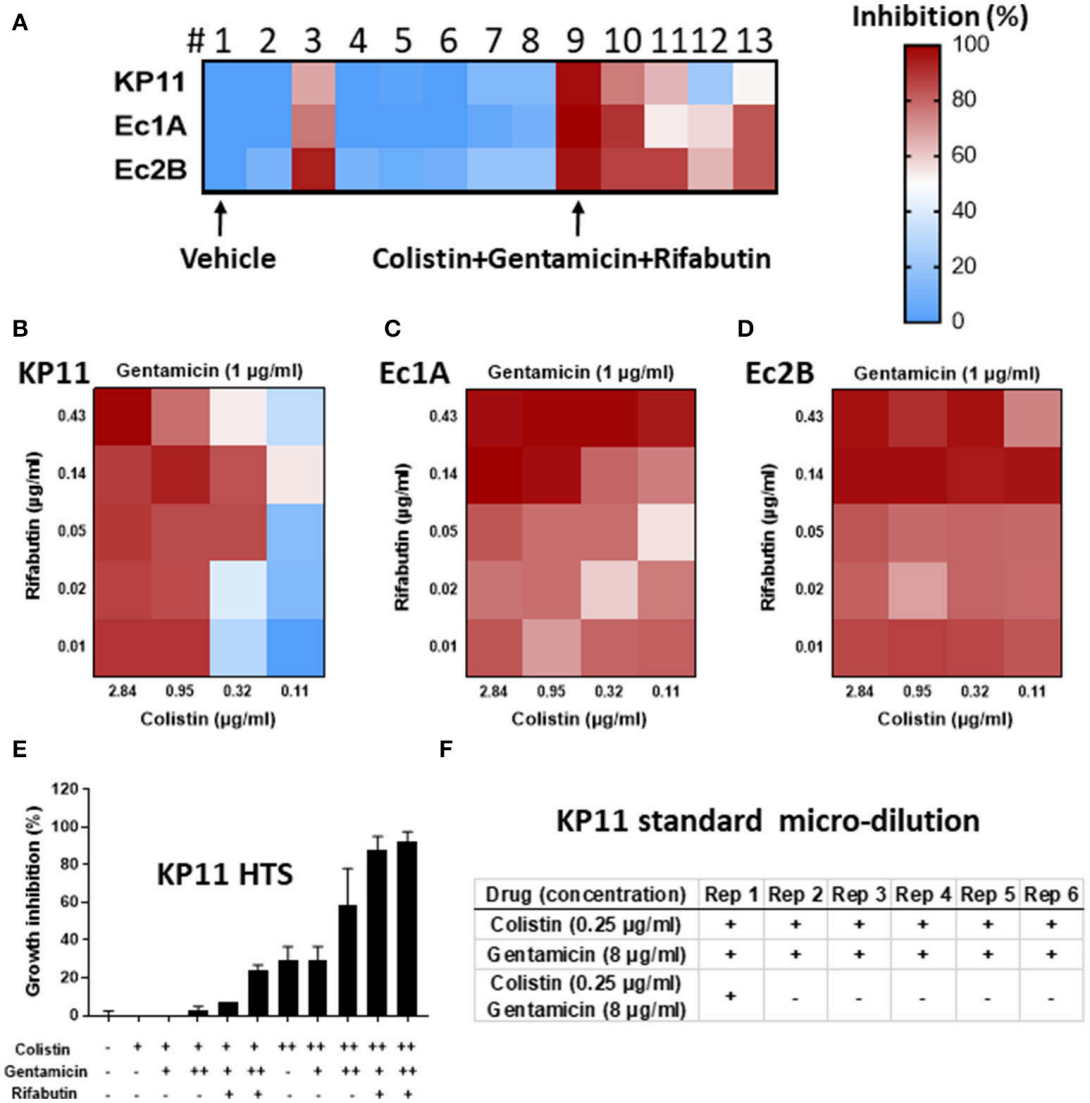
“Real-time” high-throughput drug and combination testing against multidrug-resistant organisms (MDRO). A total of 8 drugs and 14 drug combinations (in 2,304 samples) were tested against three MDRO *K. pneumoniae*-KP11, *E. coli*-Ec1A and *E. coli*-Ec2B. **(A–D)** Growth inhibition of KP11, Ec1A and Ec2B by individual drugs (1 vehicle; 2 gentamicin; 3 colistin; 4 rifabutin; 5 imipenem; 6 ceftazidime; 7 meropenem; 8 tigecycline) and drug combinations (9 colistin+gentamicin+rifabutin, 10 colistin+imipenem+rifabutin, 11 meropenem+tigecycline+colistin, 12 meropenem+tigecycline+colistin+ceftazidime, 13 meropenem+tigecycline+colistin+rifabutin) in high-throughput bacterial assay. Colors represent drug potency (% of growth inhibition). Vehicle control was normalized as 0% inhibition and no bacterium was normalized as 100% inhibition. *n* = 4, mean ± SEM. **(E)** Growth inhibition of KP11 by combinations of colistin, gentamicin, and rifabutin in high-throughput bacterial assay. Vehicle (–), colistin at 0.5 μg/ml (+) or 2 μg/ml (++), gentamicin at 1 μg/ml (+) or 4 μg/ml (++), or rifabutin at 0.06 μg/ml were tested at indicated conditions. *n* = 3, mean ± SEM. **(F)** Growth inhibition of KP11 by colistin, gentamicin, and the combination of colistin and gentamicin in standard micro-broth assay. 100 μg/ml meropenem (–) or vehicle alone (+) were included as control. + was visual bacterial growth. – was no visual bacterial growth. Rep, repeat. *n* = 6.

To identify the most suitable alternative antibiotic combinations, a “real-time” HTS combinational drug screen was performed. Three MDROs were isolated from the patient on admission: one *Klebsiella pneumoniae* (KP11) and two *Escherichia coli* (Ec1A and Ec2B). These strains were tested for sensitivity to a total of 8 drugs (gentamicin, colistin, rifabutin, imipenem, ceftazidime, meropenem, tigecycline, and auranofin) in 9 unique combinations and 14 total combinations. Each sample was tested in quadruplicate, amounting to a total of 2,304 samples which were run on three 1,536-well plates using clinically-relevant drug concentrations (Figures 1A–D). None of the tested drugs was able to completely suppress the growth of all three strains as monotherapy. Notably, colistin was the only solo drug that mediated >50% inhibition of each strain. It exerted a greater inhibitory effect on Ec1A and Ec2B than KP11. The other antibiotics demonstrated negligible activity against these three strains (<20% inhibition).

For the three-drug combinations, #9 (gentamicin+colistin+rifabutin) successfully suppressed > 90% growth of all three strains (Figure 1A). #10 (colistin+imipenem+rifabutin) and #11 (meropenem+tigecycline+colistin) were less effective, inhibiting 54–90% of all three strains. In the four-drug combination tests, #12 (meropenem+tigecycline+colistin+ceftazidime) and #13 (meropenem+tigecycline+colistin+rifabutin) inhibited 22–83% of all three strains.

Next, we studied the three-drug combination (gentamicin+colistin+rifabutin) against KP11 at various drug concentrations (Figure 1E). Colistin was tested as a single drug at 0.5 or 2 μg/ml. Either 1 or 4 μg/ml gentamicin was added into colistin as two-drug combinations. 0.06 μg/ml rifabutin was added into the mixture of colistin and gentamicin to form three-drug combinations. Addition of 4 μg/ml gentamicin into 2 μg/ml colistin improved growth inhibition from 29 ± 7% to 59 ± 20%. However, growth inhibition was not enhanced with addition of 1 μg/ml gentamicin, suggesting dose-dependent synergy. Addition of 0.06 μg/ml rifabutin into 4 μg/ml gentamicin and 2 μg/ml colistin further improved growth inhibition to 93 + 5%.

Verification of the inhibitory effect of these drug combinations in an accredited clinical laboratory by traditional methods could not be performed in real-time, prior to the decision about therapy, due to time constraints. The additional testing was performed retrospectively to validate the selected drug combinations for this report. Colistin and gentamicin as single agents and in combination with each other were tested against KP11 in the broth microdilution assay recommended by the CLSI. Neither 0.25 μg/ml colistin nor 8 μg/ml gentamicin as single therapy was fully inhibitory. The combination of 0.25 μg/ml colistin and 8 μg/ml gentamicin was inhibitory in all but one of six replicates (Figure 1F). The significance of this finding is unclear but may be related to the poor reproducibility of colistin, which is well known to interfere with methods of MIC testing (6). Additionally, a relatively low density of MDRO was inoculated in the HTS assay, while a higher density of MDRO was inoculated in the broth microdilution assay. This may contribute to the differing degree of inhibition observed for colistin between these two methods.

Infection with MDROs in this case proved difficult to treat due to the extensive drug resistance and the potential liver toxicity. We continued colistin as the “backbone” of the antibiotic regimen based on the initial MIC data generated by conventional susceptibility tests and successful clinical microbiological control. Imipenem was discontinued and replaced with piperacillin-tazobactam plus gentamicin, the former to maintain broad spectrum coverage for the infected hematoma and the latter for synergy based on the HTS data. Rifabutin was eschewed secondary to an adverse side effect profile and problematic drug-drug interactions. Beginning 2 days after the change in antibiotic therapy and continuing over the next 3 weeks, total bilirubin levels steadily declined to 2.2 mg/dl and the MDR *E. coli* and *K. pneumoniae* remained controlled with negative blood cultures, which allowed reinstatement of the patient's aplastic anemia therapy that included eltrombopag (contraindicated with severe liver toxicity) prior to transplant. The patient subsequently received a haplo-cord transplant but died 4 months after presentation due to disseminated infection with a resistant *Scopulariopsis* mold. MDROs did not appear to be a source of systemic infection at the time of his death. The patient's direct hyperbilirubinemia was, in the end, ascribed to imipenem toxicity given the tight temporal correlation between increasing/decreasing bilirubin levels and imipenem exposure, as well as the lack of convincing evidence for other etiologies.

## Discussion

Multidrug-resistance in bacteria has risen markedly, limiting effective treatment options (7). Combination antibiotic therapy has shown benefit in severe MDRO infections, reducing mortality and potentially reducing further development of resistance (8). Expanded susceptibility testing including broad-based screening for synergistic antibiotic combinations may assist clinicians in identifying not only the optimal first-line antibiotic regimen but also alternative regimens when drug interactions or toxicities occur, as was the case for this patient. This report presents a research protocol for high-throughput susceptibility testing to identify effective *in vitro* drug combinations within a clinically-actionable time frame. This model has the potential for widespread implementation at many hospitals where these infections arise and are treated, particularly the 77 academic universities or hospitals within the US that already possess HTS capability (9).

The data here are limited in that they utilize *in vitro* combination therapy testing which may lead to discrepant results (10) and lack controlled clinical trial data to evaluate effects on patient outcomes. For example, while cystic fibrosis patients are among the populations that stand to benefit the most from validated synergy testing, only one controlled clinical trial assessing its efficacy has been published (11). Although it did not find evidence of benefit from synergy testing compared to conventional susceptibility testing, a recent Cochrane Review on the subject (which identified only one trial—the trial referenced above—which was suitable for inclusion in its analysis) lamented the extreme paucity of evidence available for assessment (12). A multi-center randomized controlled study is currently underway to assess the benefits of colistin in combination with carbapenems vs. colistin alone based on promising *in vitro* synergy data; however, this will only indirectly address the question of whether combination susceptibility testing has clinical utility (13). Clearly more study is required in this area as combination therapy will only become more common in the foreseeable future.

The other conclusion to be drawn from this case is that HTS technology has developed to the point of becoming tractable for a wide range of applications. With its miniaturized format and automated workflow, the expanded susceptibility testing performed in the case requires relatively scant raw materials and human labor on a per-assay basis. Therefore, if data on pathogen sensitivity to combinations of antimicrobials are determined to have clinical value, HTS will serve as a powerful vehicle for its actualization.

## Ethics statement

Written informed consent was obtained from the patient representative for publication of the aforementioned data. All patients cared for in the NIH Clinical Center are in IRB approved research protocols with signed consents.

## Author contributions

PW, WZ, and WS: Conception and design of the work; WS, SH, MX, RC: Data collection; WS, SH, WZ, and PW: Data analysis and interpretation, manuscript writing, and critical revision of the article; WS, SH, MX, RC, WZ, and PW: Approval of the final version of the article.

### Conflict of interest statement

The authors declare that the research was conducted in the absence of any commercial or financial relationships that could be construed as a potential conflict of interest.
